# Operant down-conditioning of the soleus H-reflex in people after stroke

**DOI:** 10.3389/fresc.2022.859724

**Published:** 2022-07-22

**Authors:** Aiko K. Thompson, Christina R. Gill, Wuwei Feng, Richard L. Segal

**Affiliations:** ^1^Department of Health Sciences and Research, College of Health Professions, Medical University of South Carolina, Charleston, SC, United States; ^2^Department of Neurology, College of Health Professions, Duke University School of Medicine, Durham, NC, United States; ^3^Department of Rehabilitation Sciences, College of Health Professions, Medical University of South Carolina, Charleston, SC, United States

**Keywords:** spinal cord plasticity, operant conditioning, learning, spasticity, hemiparesis

## Abstract

Through operant conditioning, spinal reflex behaviors can be changed. Previous studies in rats indicate that the sensorimotor cortex and corticospinal tract are essential in inducing and maintaining reflex changes induced through conditioning. In people with incomplete spinal cord injury (SCI), an operant down-conditioning protocol decreased the soleus H-reflex size and improved walking speed and symmetry, suggesting that a partially preserved spinal cord can support conditioning-induced plasticity and benefit from it. This study examined whether down-conditioning can decrease the soleus H-reflex in people with supraspinal injury (i.e., cortical or subcortical stroke). Operant down-conditioning was applied to the soleus H-reflex in a cohort of 12 stroke people with chronic spastic hemiparesis (>12 months from stroke onset of symptoms). Each participant completed 6 baseline and 30 conditioning sessions over 12 weeks. In each baseline session, 225 control H-reflexes were elicited without any feedback on H-reflex size. In each conditioning session, 225 conditioned H-reflexes were elicited while the participant was asked to decrease H-reflex size and was given visual feedback as to whether the resulting H-reflex was smaller than a criterion value. In six of 12 participants, the conditioned H-reflex became significantly smaller by 30% on average, whereas in other 6 participants, it did not. The difference between the subgroups was largely attributable to the difference in across-session control reflex change. Ten-meter walking speed was increased by various extent (+0.04 to +0.35, +0.14 m/s on average) among the six participants whose H-reflex decreased, whereas the change was 0.00 m/s on average for the rest of participants. Although less than what was seen in participants with SCI, the fact that conditioning succeeded in 50% of stroke participants supports the feasibility of reflex down-conditioning in people after stroke. At the same time, the difference in across-session control reflex change and conditioning success rate may reflect a critical role of supraspinal activity in producing long-term plasticity in the spinal cord, as previous animal studies suggested.

## Introduction

Operant conditioning of a spinal reflex induces the targeted plasticity in a specific reflex pathway. Forty years of basic science studies have revealed the presence of far-reaching spinal cord plasticity associated with reflex operant conditioning and the role of the brain in producing and maintaining this plasticity [reviewed in (1–7)].

In the spinal cord, successful down-conditioning of the soleus H-reflex, an electrical analog of the spinal stretch reflex, reduces axonal conduction velocity and increases the firing threshold in motoneurons (8–10), and together with a small decrease in the primary afferent excitatory post-synaptic potential (EPSP), these changes largely explain the resulting smaller H-reflex. With down-conditioning, sodium channels on motoneurons (11, 12) as well as several other synaptic populations on motoneurons change (8, 9, 11, 13–15); GABAergic terminals increase in number (14), accompanied by a corresponding increase in the number of identifiable GABAergic interneurons in the ventral horn (12). Reflex conditioning even affects the contralateral side of the spinal cord (16, 17). In summary, H-reflex conditioning produces complex multi-site plasticity in the spinal cord (5).

The role of supraspinal plasticity in inducing and maintaining spinal cord plasticity reflected in the H-reflex change has been discovered through a series of controlled lesion studies (reviewed in (7)). The corticospinal tract (CST) (not other descending or ascending pathways) and sensorimotor cortex are most immediately needed for conditioning-induced H-reflex changes to occur (18–21). Spinal cord plasticity can be sustained by the plasticity in sensorimotor cortex for many days (20, 21), while the long-term maintenance of such cortical plasticity depends on the cerebellum (22, 23) and its plasticity. Furthermore, cerebellar plasticity is guided and maintained by climbing fiber input from the inferior olive (24). In short, the cumulating observations in animal studies indicate that a hierarchy of CNS plasticity is required for induction and maintenance of conditioning-induced spinal cord plasticity.

Currently, in humans, how much of each structure needs to be available (preserved) for induction and maintenance of this complex plasticity remains unknown. In people with incomplete spinal cord injury (SCI), an operant down-conditioning protocol can decrease the soleus H-reflex size (25). This suggests that the remaining CNS with preserved CST after incomplete SCI is still capable of supporting the conditioning-induced spinal cord plasticity. In post-stroke humans, unlike the rats after sensorimotor cortex ablation (20), stroke-caused supraspinal injuries are most likely partial injuries. Thus, different from the previous rat study in which down-H-reflex conditioning was not possible at all after sensorimotor cortex ablation (20), reflex operant conditioning may be possible in at least some of the people after stroke. Here, we hypothesized that in humans the CNS after supraspinal injury (i.e., cortical or subcortical stroke) can permit the reflex conditioning-induced plasticity to occur, at least in some cases. In the present study, operant down-conditioning was applied to the soleus H-reflex in individuals after stroke with chronic plantarflexor spasticity. This sub-population of stroke survivors was selected, for its relative similarity in sensorimotor deficit to the previously studied sub-population of individuals with chronic incomplete SCI (25, 26). In a long history of reflex operant conditioning research [reviewed in (1–7)], this is the first study to examine the possibility of H-reflex conditioning in human post-stroke population.

## Materials and methods

### Participants

Thirteen adults (37–76 years old) with a history of cortical or subcortical stroke and chronic spastic hemiparesis were enrolled into this study. One stopped taking baclofen during study period, and thus, he was excluded in the data analysis. The rest of 12 participants' profiles are summarized in Table 1. Location of stroke was basal ganglia for participants S05 and S09; all others had cortical stroke. Prior to participation, all participants gave informed consent by themselves, which was reviewed and approved by Helen Hayes Hospital and the Medical University of South Carolina Institutional Review Board. For each prospective participant, was determined by a vascular neurologist (WF). Inclusion criteria were: (1) >1 year post stroke; (2) neurologically stable for >3 months prior to participation; (3) medical clearance to participate (with the expectation of no change in medication for >3 months); (4) ability to ambulate with or without an assistive device (except parallel bars) at least 10 meters; and 5) unilateral plantarflexor spasticity (hemiparesis). For each participant, the investigator AKT confirmed the induction of clonus by single pulse tibial nerve stimulation (i.e., H-reflex eliciting stimulation) during standing. Note that histories of botulinum toxin treatments did not preclude a study candidate's participation, as long as more than 3 months had passed since the last treatment and there was no plan to receive another dose during the candidate's study participation. Exclusion criteria were (1) motoneuron injury; (2) history of myocardial infarction or congestive heart failure, pacemaker use; (3) a medically unstable condition including uncontrolled diabetes with recent weight loss, diabetic coma, or frequent insulin reactions; and (4) cognitive impairment affecting their ability of informed consent or successful completion of the protocol.

**Table 1:** Clinical demographics of study participants.

**ID**	**Age (yr)**	**Sex**	**Post stroke (month)**	**Type** ^1^	**Etiology**	**Baseline Hmax**^2^ **(%M**_max_**)**	**Initial 10-m** **speed (m/s)**	**Final H-reflex size**^3^ **(%baseline)**
S10^*^	76	M	18	H	Small vessel disease	61.5	N/A	46.1
S13^*^	58	M	38	I	Undetermined	56.4	0.5	48.2
H03^*^	37	F	30	I	Undetermined	82.2	0.38	75.6
S12^*^	50	M	33	I	Undetermined	59.8	1.30	79.7
H02^*^	40	F	22	I	Carotid artery dissection	86.9	0.75	81.8
S05^*^	62	M	38	H	Small vessel disease	54.4	0.77	86.2
S06	48	F	38	I	Undetermined	87.4	0.17	95.1
S04	64	F	23	I	Undetermined	34.8	0.60	98.5
H01	46	F	28	H	Cerebral aneurysm	44.6	1.25	100.5
S02	69	M	54	I	Atherosclerosis	53.0	0.74	105.6
S11	40	M	138	H	Cerebral aneurysm	66.5	0.36	109.2
S09	60	F	69	I and H	Small vessel disease	23.6	0.34	117.7

* *Individuals in whom H-reflex conditioning was successful (i.e., conditioned H-reflex size of the last 6 sessions was significantly different from the six baseline sessions (p < 0.05 by U-test))*.

1 *Type of stroke: ischemic (I) or hemorrhagic (H)*.

2 *Baseline H_max_ size was calculated as the average H_max_ size for the 6 baseline sessions, expressed in %M_max_*.

3 *Final H-reflex size was calculated as the average conditioned H-reflex size for conditioning sessions 28-30*.

### Study overview

**Figure 1:**
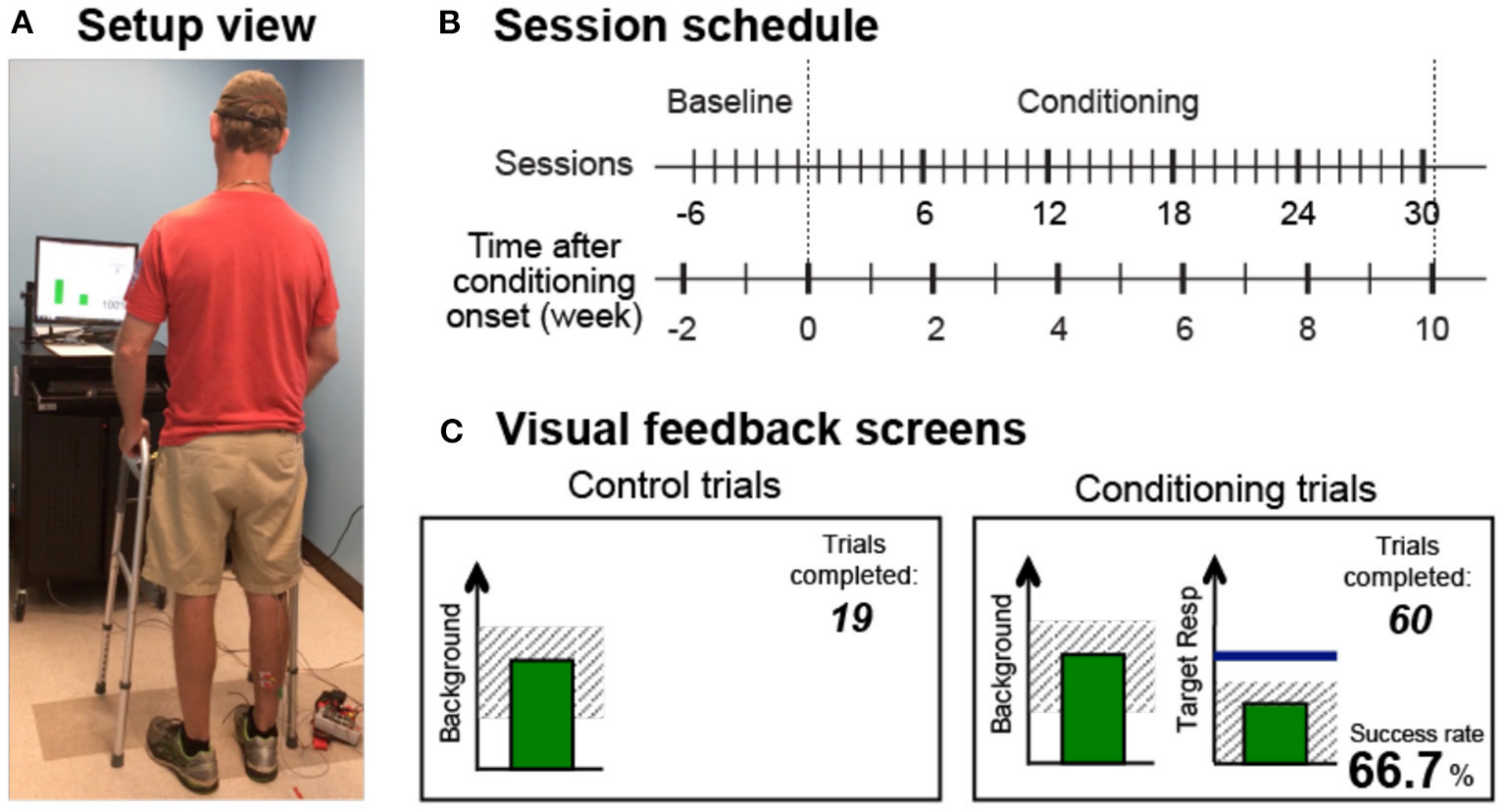
Reflex conditioning session setup overview. **(A)** Setup view. Soleus H-reflexes were elicited while the participant maintained a stable standing posture and the soleus and TA muscle activity. **(B)** Session schedule. Each participant was exposed to six baseline sessions and 30 conditioning sessions that occurred at a pace of 3 times per week. **(C)** Visual feedback screens for control and conditioning trials. During all trials, the number of trials completed within its block is displayed, and the background EMG panel shows the correct range (shaded) and the current value (green vertical bar, updated every 200 ms). If the soleus EMG stays in the correct range for at least 2s, the tibial nerve is stimulated, and an H-reflex is elicited. In control trials (left), H-reflex size is not shown. In conditioning trials (right), the shading in the Target Response panel indicates the rewarded H-reflex size range for down-conditioning. The dark horizontal line indicates the average H-reflex size for the 6 baseline sessions, and the vertical bar shows the size of the most recent H-reflex trial. If that H-reflex size falls into the shaded area, the bar becomes green, and the trial is a success. If it falls out of the shaded area, the bar is red and the trial is a failure. The running success rate for the current block is also shown.

The operant conditioning protocol used in this study was the same as the one used in individuals after SCI (25). In several preliminary sessions, the participant was familiarized with the protocol and appropriate background EMG and M-wave criteria were defined. In each session, the soleus H-reflex was elicited while the participant maintained a natural standing posture (Figure 1A) and a stable level of soleus and tibialis anterior (TA) background EMG. For all participants, the M-wave size for H-reflex trials was chosen from the rising phase of the H-reflex recruitment curve (typically with the stimulus at just above M-wave threshold). After preliminary sessions, each participant completed six baseline sessions and 30 conditioning sessions that occurred at a pace of three times per week. To prevent the normal diurnal variation in H-reflex size (27–30) from affecting the results, a participant's sessions always occurred at the same time of day (i.e., within the same 3-h time window). A typical session took about 1 h.

In each baseline session, 225 control H-reflexes were elicited without any feedback on H-reflex size. In each conditioning session, 20 control H-reflexes were elicited as in the baseline sessions and then 225 conditioned H-reflexes were elicited. In these 225 conditioning (down-conditioning) trials, the participant was asked to decrease H-reflex size and was given visual feedback after each stimulus to indicate whether the resulting H-reflex was smaller than a criterion value. As detailed in the following sections, background EMG and M-wave size were kept stable throughout data collection.

Before the baseline and after 30 conditioning sessions, 10-meter walk test was performed. The participant was instructed to walk at his/her fastest comfortable speed from the 0-m marker to the 14-m marker, and the speed was calculated for the 10-m distance between the 2- and 12-m markers. For each participant, the use of an assistive device (e.g., cane and walker) for this test was maintained the same for both before and after conditioning. For each assessment, three trials were averaged together to calculate the 10-m speed.

### Session protocol and EMG recording

At the beginning of each session, EMG recording and stimulating electrodes were placed over the lower leg. Soleus and TA EMG signals were recorded with surface self-adhesive Ag-AgCl electrodes (2.2 × 3.5 cm, Vermed, Inc., Bellows Falls, VT). EMG activity was amplified, band-pass filtered (10–1,000 Hz), sampled at 3,200 Hz, and stored. To elicit the soleus H-reflex, the tibial nerve was stimulated in the popliteal fossa, using surface Ag-AgCl electrodes (2.2 × 2.2 cm for cathode, 2.2 × 3.5 cm for anode, Vermed, Inc.) and isolated constant current stimulation (Grass S48 stimulator with an SIU-5 stimulation isolation unit and a CCU1 constant current unit, Natus Neurology, West Warwick, RI). The stimulating electrode pair were placed so as to minimize the H-reflex threshold and to avoid stimulation of other nerves. For eliciting the H-reflex and M-wave, a 1-ms square stimulus pulse was delivered when the participant had maintained soleus EMG activity within a pre-determined range (i.e., one's natural standing level, which is ~10%−20% of maximum voluntary contraction level in individuals without neurological injuries (31)) and TA EMG at a resting level (typically <7–8 μV) for at least 2 s. The minimum interstimulus interval was 5 s.

An H-reflex – M-wave recruitment curve was obtained while the standing participant maintained the above mentioned pre-determined level of soleus EMG activity. Stimulus intensity was varied in increments of 1.2–2.5 mA from soleus H-reflex threshold to the maximum H-reflex (H_max_) to an intensity just above what was needed to elicit the maximum M-wave (M_max_) (32–34). About 10 different intensities were used to obtain each recruitment curve, and four EMG responses were averaged to measure the H-reflex and M-wave at each intensity. After the H-M recruitment curve measurement, the session continued by following the protocol of a baseline session or a conditioning session (see below). For all trials in all sessions, H-reflexes were obtained in the same natural standing posture and were accompanied with a pre-determined size of M-wave (i.e., just above M-wave threshold).

In the baseline sessions, the H-reflex – M-wave recruitment curve was followed by three blocks of 75 control trials in which the participant was not asked to change H-reflex size and was not given visual feedback as to H-reflex size. In the conditioning sessions, the H-reflex – M-wave recruitment curve was followed by a 20-trial block of “within-session” control H-reflex trials identical to those of the baseline sessions. This was followed by three blocks of 75 conditioned H-reflex trials (i.e., 225 trials in total), in which the participant was asked to decrease H-reflex size and was provided with immediate visual feedback that indicated his or her success in doing so (see the next section).

In order to avoid session-to-session variability in the location of stimulating and recording electrodes, the positions of all electrodes are mapped in relation to landmarks on the skin (e.g., scars or moles) during the first preliminary session. These measures are used to place the electrodes in all subsequent sessions. For each participant, the same investigator placed the electrodes for all study sessions.

### Visual feedback

Figure 1C shows the visual feedback provided to the participant during H-reflex trials. The screen presented two graphs, one for soleus background EMG activity (left) and one for H-reflex size (right). The background EMG panel was the same for both control and conditioned H-reflex trials. If the participant had kept the background EMG bar in the specified range for 2 s and at least 5 s had passed since the last stimulus, a stimulus pulse elicited the H-reflex and M-wave. The feedback screen differed for control and conditioned H-reflex trials. For the control trials, the H-reflex graph was not shown. For the conditioned trials, a vertical bar reflecting H-reflex size [i.e., the average rectified EMG in the H-reflex interval (typically around 35–50 ms after the stimulus)] appeared 200 ms after the stimulus. The bar was green only if H-reflex size met the reward criterion (i.e., falling into the shaded rewarded range, indicating success), and the bar was red if H-reflex size did not satisfy the criterion (i.e., getting out of the rewarded range, indicating failure). The reward criterion for each block was determined based on the average and distribution of H-reflex sizes across trials from the previous block of trials. That is, the criterion was selected such that if the distribution of H-reflex sizes for the new block *were* similar to that for the previous block, 50%−60% of the trials would be successful (35). In each conditioning session, the criterion value for the first block of 75 conditioned H-reflex trials was determined based on the immediately preceding block of 20 control trials, and the criterion values for the second and third conditioned blocks were based on the immediately preceding block of 75 conditioned trials. For each block, the participant earned a modest extra monetary reward if the success rate exceeded 50% (see (31) for full details of protocol).

### H-reflex data analysis

For each session of each participant, two measures of H-reflex sizes were calculated. Regardless of whether it was for a baseline session or for a conditioning session, for each session, H-reflexes from all three 75-trial blocks were averaged together and called “conditioned H-reflex,” and H-reflexes from the first 20 control trials (i.e., the first 20 trials of the first block of 75 control trials for baseline sessions and 20 within-session control trials for conditioning sessions) were averaged together and called “control H-reflex.” Since these control H-reflexes were elicited without feedback on H-reflex size (see the left panel of Figure 1C) and outside of the conditioning paradigm, the measurement of control H-reflex served two purposes. First, for a given session, it served as the within-session control H-reflex to the conditioned H-reflex. Second, its change across sessions served as a marker of long-term plasticity in the H-reflex pathway that would be present outside of the conditioning paradigm (31). In addition, for each participant's each session, the within-session difference between the conditioned and control H-reflexes was calculated. Since this within-session reflex change reflected the reflex size reduction that the participant learned to produce in response to operant down-conditioning feedback and instructions (i.e., in a task-dependent manner), this measure was also called “task-dependent adaptation” (31).

For these calculations, H-reflex size was defined as average rectified EMG amplitude in the H-reflex interval minus average soleus background EMG (i.e., rectified EMG amplitude in the prestimulus 50-ms period). Changes in these H-reflex sizes across sessions were quantified in percent of their average values for the six baseline sessions. We also determined for each participant the final effect of conditioning on the conditioned H-reflex by averaging the conditioned H-reflexes of conditioning sessions 28–30 and expressing the result in % of the average conditioned H-reflex of the six baseline sessions. (Thus, a value of 100% indicates no change in H-reflex size.) The final effect of conditioning on the control H-reflex was calculated similarly by averaging the control H-reflexes of conditioning sessions 28–30 and expressing the result in % of the average control H-reflex of the six baseline sessions (see (31)).

### Statistical analysis

In this study, we attempted to examine the hypothesis that reflex operant conditioning can change the soleus H-reflex size in the direction of conditioning in some of the people after supraspinal injury (i.e., cortical or subcortical stroke). We completed the conditioning protocol in 12 individuals after stroke, to examine if and in how many of them H-reflex down-conditioning was possible (which was assessed as whether the conditioned H-reflex in the conditioning sessions 25–30 were smaller than those in six baseline sessions).

To determine for each participant whether H-reflex down-conditioning was successful (i.e., whether H-reflex became smaller through down-conditioning), the average conditioned H-reflexes of the final six conditioning sessions were compared to the average H-reflexes of the six baseline sessions by Mann–Whitney *U*-test. Then, according to its result, the individual whose conditioned H-reflexes from the final six conditioning sessions were significantly smaller than those of the six baseline sessions (*p* < 0.05, one-tailed), was sorted into the Ss group. The individuals, whose H-reflexes did not differ between the baseline and the final six conditioning sessions or whose H-reflexes from the final six conditioning sessions were not smaller, were sorted into the Sns group in the analysis process. To assess whether age, stroke chronicity (i.e., post-stroke duration in months), pre-conditioning walking ability (i.e., 10-m walking speed), and baseline H-reflex amplitude affected H-reflex down-conditioning results, these parameters were compared between the Ss and Sns groups.

To examine the effect of conditioning across multiple sessions, a repeated measures ANOVA was used to evaluate conditioned and control H-reflex sizes and within-session reflex change across successive six-session blocks (i.e., baseline sessions 1–6 and conditioning sessions 1–6, 7–12, 13–18, 19–24, and 25–30). We also assessed over all sessions the stability of the soleus M_max_ and the TA and soleus background EMG levels, and the H_max_ using a repeated measures ANOVA, similar to the evaluation of the group effects on H-reflex sizes (31).

To test for functional change, paired *t*-test was used for comparing 10-m walking speeds before and after H-reflex conditioning.

## Results

### Background EMG, M-wave, and participant profiles

All 12 participants completed six baseline sessions and 30 conditioning sessions. H-reflex down-conditioning was successful (i.e., the average conditioned H-reflexes for conditioning sessions 25–30 were significantly less than those for the six baseline sessions (31)) in six of the participants. Final H-reflex sizes for all 12 participants are listed in Table 1. As anticipated from the protocol and previous studies (25, 31, 36), the soleus M_max_ and M-wave sizes in control and conditioning trials and the soleus and TA background EMG remained stable across all sessions in the individuals whose H-reflex decreased significantly (Ss group) and in the individuals whose H-reflex did not decrease (Sns group). Note that across both groups of participants, the M-wave that accompanied control and conditioning H-reflexes was 5 ± 3 (mean ± SD) %M_max_ in amplitude, which was 48 ± 21 μV in mean rectified value. Two-way repeated measures ANOVA (group × session block) showed non-significant effects of group [*F*_(1, 50)_ = 0.02–2.32, *p* = 0.90–0.16], session block [*F*_(5, 50)_ = 0.76–2.13, *p* = 0.58–0.08], and group × session block [*F*_(5, 50)_ = 0.43–1.15, *p* = 0.83–0.35]. The soleus H_max_ also did not differ significantly between the groups [*F*_(1, 50)_ = 0.03, *p* = 0.87] or session blocks [*F*_(5, 50)_ = 0.53, *p* = 0.76], and there was no interaction between the groups and session blocks [*F*_(5, 50)_ = 1.02, *p* = 0.42]. Overall, these results support the stability of nerve stimulation and EMG recording across sessions and in both Ss and Sns groups of individuals.

Next, to assess a possibility that certain characteristics of participants may have affected H-reflex down-conditioning results, age, stroke chronicity (i.e., post-stroke duration in months), and pre-conditioning walking ability (i.e., 10-m walking speed) were compared between the groups; there was no between-group differences in these parameters (*p* = 0.81, 0.15, and 0.20 by two-tailed *U*-test, respectively). To assess whether pre-conditioning H-reflex size affected conditioning results, we compared the baseline soleus H_max_ between the groups [67 ± 14% M_max_ (which equals to 3.9 ± 1.4 mV peak-to-peak amplitude) for Ss; 52 ± 23%M_max_ (which equals to 3.0 ± 2.2 mV) for Sns]; and it did not differ significantly (*p* = 0.17). Also, there was no correlation between the baseline soleus H_max_ amplitude and final H-reflex size (*r* = −0.35), or between the stroke chronicity and the baseline soleus H_max_ amplitude (*r* = −0.20). Altogether, these results indicate that it is unlikely that most obvious participant characteristics such as, age, chronicity, and H-reflex size, were strong predictors or determinants of H-reflex down-conditioning results in people after stroke.

### H-reflex changes in the individuals whose H-reflex decreased

**Figure 2:**
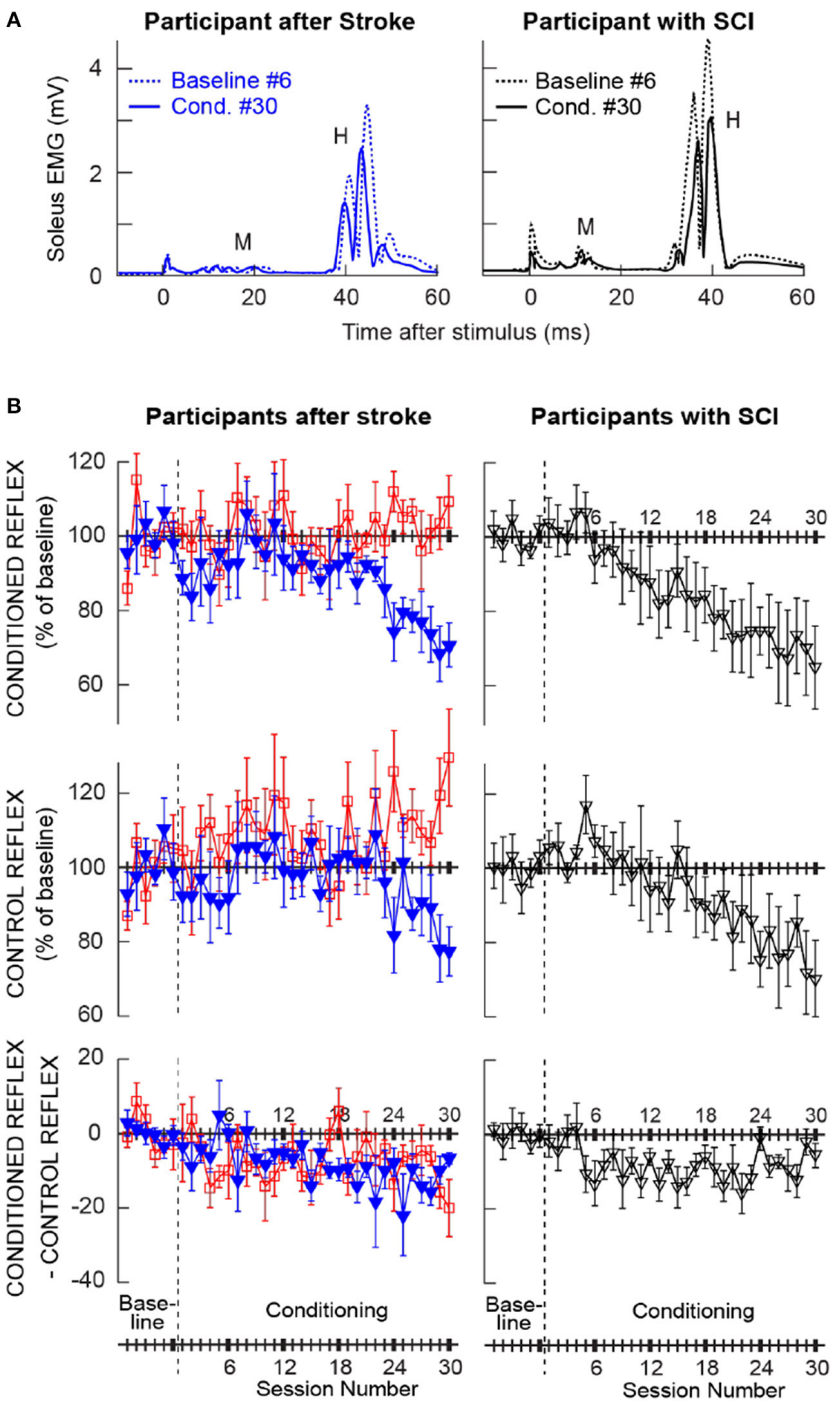
Changes in the soleus H-reflex brought about by operant down-conditioning. **(A)** Average conditioned H-reflexes in a baseline session (dashed line) and the last conditioning session (solid line) from a participant after stroke (left) and a participant with chronic incomplete spinal cord injury (right) (25) whose H-reflex decreased significantly. A small stimulus artifact is present at time 0. 225 trials are averaged together for each sweep. **(B)** Average (±SE) H-reflex values for baseline and conditioning sessions for the present groups of participants after stroke (left column) and for the previous group of participants with SCI [right, from (25)]. The present participants after stroke are separated into two groups: the ones whose conditioned H-reflex size decreased significantly (blue, Ss group, *N* = 6) and the ones whose conditioned H-reflex size did not decrease (red, Sns group, *N* = 6). Top: Average conditioned H-reflex size. Middle: Average control H-reflex size. Bottom: Average of conditioned H-reflex size minus control H-reflex size [i.e., task-dependent adaptation; for details see (31)].

Figure 2A shows the averaged H-reflex sweeps from the sixth baseline session and the thirtieth conditioning session from a participant after stroke (left) and a participant with chronic SCI (right, from (25)), whose H-reflex decreased significantly through down-conditioning. For each sweep, 225 H-reflex sweeps (i.e., H-reflexes from the three blocks of 75 trials) were averaged together. These reflexes (i.e., conditioned H-reflexes) became much smaller after down-conditioning. As noted above, M-wave size did not change across the sessions.

Left column of Figure 2B shows the average courses of H-reflex change for the present groups of participants after stroke; ones whose H-reflex decreased significantly (i.e., Ss group, in blue) and the ones whose H-reflex did not decrease (i.e., Sns group, in red). Right column of Figure 2B shows the average courses for the group of down-conditioned participants with SCI from the earlier study (25), for visual comparison. These panels show the time courses of changes for the conditioned H-reflex (top), the control H-reflex (middle), and the within-session change between the conditioned and control H-reflexes (bottom). The control H-reflex is the H-reflex for the first 20 trials of each baseline or conditioning session in which the participant was not asked to decrease the H-reflex and was not provided with feedback as to reflex size. The within-session difference reflects the task-dependent adaptation that the participants produce when they are asked to decrease H-reflex size (31). Table 2 summarizes the changes in H-reflex size over the course of 30 down-conditioning sessions in successive six-session blocks.

**Table 2:** Changes in the soleus H-reflex with down-conditioning during standing in the present groups of individuals after stroke whose H-reflex decreased significantly (Ss) and whose H-reflex did not decrease (Sns), and individuals with SCI from (25).

	**C1–6 (%)**	**C7–12 (%)**	**C13–18 (%)**	**C19–24 (%)**	**C25–30 (%)**
Conditioned reflex
Ss	88.9 ± 7.2	98.4 ± 2.0	90.4 ± 4.5	85.6 ± 3.7	73.2 ± 6.5^*^
Sns	98.3 ± 4.2	105.8 ± 4.7	96.5 ± 5.2	102.7 ± 3.3	103.6 ± 2.1
SCI	102.1 ± 4.9	92.1 ± 6.2	84.5 ± 8.2	75.4 ± 7.6 ^*^	69.7 ± 11.4^*^
Control reflex
Ss	93.0 ± 6.5	104.0 ± 4.9	99.2 ± 4.7	98.1 ± 5.3	87.2 ± 6.3
Sns	104.7 ± 3.9	114.1 ± 6.7^*^	101.6 ± 4.7	111.3 ± 3.2	115.5 ± 5.0^*^
SCI	103.5 ± 3.8	100.6 ± 5.7	94.8 ± 6.9	85.5 ± 5.4	77.4 ± 9.4^*^
Within-session change
Ss	−4.0 ± 2.8	−5.5 ± 3.8	−8.8 ± 6.9	−12.5 ± 4.4^*^	−14.0 ± 3.3^*^
Sns	−6.4 ± 3.7	−8.3 ± 3.4	−5.1 ± 3.7	−7.7 ± 3.7	−10.1 ± 2.8
SCI	−1.4 ± 6.2	−8.4 ± 2.8	−10.3 ± 3.5	−10.0 ± 3.3	−7.7 ± 2.8

*All values are expressed in % of baseline value (mean ± SE)*.

* *Significant differences from the 6 baseline sessions (p < 0.05, Dunnett's test for post hoc)*.

These courses of H-reflex changes are different from those observed previously in people with SCI, who were exposed to the same protocol. For the Ss group, while the final average conditioned H-reflex size (i.e., the average of the last three conditioning sessions) was 70 ± 7 (mean ± SE) % of the baseline value, nearly identical to that in people with incomplete SCI (69 ± 11%, (25)), the within-session decrease was more prominent in the later phase of conditioning (−14% in people after stroke vs. −8% in people with SCI for conditioning sessions 25–30, see Table 2) and the control H-reflex decrease seemed to start only around conditioning session 24.

### H-reflex changes in the individuals whose H-reflex did not decrease

The time courses of H-reflex changes for the present subgroup of individuals whose H-reflex did not decrease (i.e., Sns group) are shown in red in Figure 2B (left column) and summarized in Table 2. In this group, the conditioned H-reflex (top) did not decrease; the control H-reflex (middle) showed paradoxical increase around conditioning sessions 7–12 (114 ± 7% of baseline, *p* < 0.05) and 25–30 (115 ± 5% of baseline, *p* < 0.05); and all the while, these individuals produced some within-session decrease for the most of conditioning nearly as much as those by individuals with SCI (25). It appears that individuals of the Sns group did not necessarily fail to decrease H-reflex size within session, but in response to H-reflex down-conditioning, the long-term across-session control reflex change occurred in the opposite direction (i.e., increase in H-reflex size). As a result, the sum of within-session change and across-session change turned out to be not negative.

### Walking speed

Individual participants' pre-conditioning walking speeds are included in Table 1. In two participants, 10-m walk test results were not available. Of the remaining 10 participants, 10-m walking speed was increased by 0.14 ± 0.13 (mean ± SD) m/s (ranged from +0.04 to +0.35 m/s) among the individuals whose H-reflex decreased significantly (i.e., Ss group; *N* = 5, *p* < 0.05, paired *t*-test). Two of those individuals (S13 and H02) had the walking speed improvement of >0.2 m/s. The walking speed did not change consistently (i.e., changed by 0.00 ± 0.05 m/s) among the individuals whose H-reflex did not decrease (i.e., Sns group; *N* = 5, *p* = 0.47). No participant changed the use of an assistive device in their daily lives over the course of study. When the relation between the final conditioned H-reflex size and the extent of walking speed change (in m/s) was examined across the 10 participants, they were negatively correlated (*r* = −0.85, *p* < 0.01); that is, participants with larger decreases in the conditioned H-reflex size had larger gain in walking speed.

## Discussion

In this study, we investigated operant down-conditioning of the soleus H-reflex (reviewed in (6, 7)) in individuals after stroke for the first time. We found that people with chronic stroke are able to reduce the soleus H-reflex size through down-conditioning, although at a lower success rate (50%) compared to the rates in people with incomplete SCI (67%−86% (25, 26)) or people with no known neurological conditions (88%−100% (31, 36, 37)). This conditioning success rate of 50% has provided an unexpected opportunity for preliminarily examining the time course of reflex changes in two subgroups of participants with chronic stroke: the individuals whose H-reflex size decreased and the individuals whose H-reflex size did not decrease. (In the previous human studies, for higher conditioning success rates and small numbers of individuals whose conditioning was unsuccessful, the analyses were focused on the data from the ones whose conditioning was successful.) Here in the sections below, we discuss the feasibility of reflex operant conditioning and the extent and time course of reflex changes that reflect spinal plasticity in people after stroke.

### H-reflex down-conditioning is possible in people after stroke

H-reflex down-conditioning was successful in six of 12 individuals of different ages with cortical (*N* = 5) and subcortical (*N* = 1) stroke (Table 1). This conditioning success rate is much better than that was found in rats with contralateral sensorimotor cortex (cSMC) lesion (20); none of the nine cSMC rats exposed to down-conditioning decreased H-reflex size, and in six of nine, the H-reflex became significantly larger over the course of down-conditioning. This difference in conditioning success rate may partially be due to the difference in the volume of remaining SMC or SMC – CST – spinal cord connection between the rats with experimental cSMC lesion and the human participants after stroke. It is possible that in rats with far weaker CST connection from the SMC to the spinal cord than humans to begin with (38), cSMC lesion completely eliminated SMC – spinal cord connection, which presumably led to 100% unsuccessful down-conditioning. In contrast, in human participants, there would be some remaining SMC – CST – spinal cord connection after stroke, although partially damaged or lost. This would permit operant-conditioning-induced spinal plasticity that relies on the SMC/CST activity (19, 20) to occur, at least in some.

As noted above, between the ones whose H-reflex became smaller and the ones whose H-reflex did not, there is no apparent difference in the basic profiles (see Table 1). For the participants of this study, no data to estimate the volume of preserved SMC or CST were available. Thus, based on the information presently available, we would not be able to further speculate the reason(s) for why the conditioned H-reflex size decreased in a half and did not in the other half. Still, the critical fact remains; that is, down-conditioning succeeded in 50% of the present participants after stroke, and this supports the feasibility of H-reflex operant down-conditioning in this population.

### Time course and composition of H-reflex changes in people after stroke

In the subgroup of individuals whose H-reflex size decreased over the course of 30 down-conditioning sessions (i.e., Ss group), the final conditioned H-reflex size was 70% of the baseline value, nearly identical to that in people with incomplete SCI (i.e., 69%, (25)). In these individuals, the within-session reflex change between the control H-reflex and the conditioned H-reflex (i.e., task-dependent adaptation) was more prominent in the later phase of conditioning (−14% in people after stroke vs. −8% in people with SCI for conditioning sessions 25–30, see Table 2); in parallel, the control H-reflex started to decrease around conditioning session 24 (Figure 2B), which is 6–7 conditioning sessions behind the course observed in people with SCI (25). These subtle differences in the time course and composition of H-reflex changes between the CNS after supraspinal injury (i.e., cortical or subcortical stroke) and the CNS after spinal injury with partially spared CST indicate that the injury location does affect the reflex conditioning-induced plasticity in people. Supraspinal injury that presumably spared some SMC – CST – spinal cord connection would not prevent task-dependent adaptation (i.e., within-session reflex decrease), which is thought to reflect immediate change in cortical influence over the excitability of the H-reflex pathway (31), but significantly delay the onset of across-session long-term change in the control H-reflex, which reflects spinal cord plasticity (31). This may further suggest that the cortical activity, more than its conduit to the spinal cord, CST, is critical in inducing long-term spinal cord plasticity.

Aside from the delayed onset of long-term spinal cord plasticity, perhaps, the most unexpected findings from this study are the within-session reflex change and the control H-reflex change in the subgroup of individuals whose conditioned H-reflex size did not decrease (Figure 2B). In these individuals (i.e., Sns group), task-dependent adaptation (i.e., within-session reflex decrease) was present throughout (i.e., to a similar extent to that in people with SCI) while the control H-reflex size increased; hence, the net result is no decrease in the conditioned H-reflex size. Therefore, it would be incorrect to label these individuals as “non-responders,” since they were reducing H-reflex size in response to operant down-conditioning during conditioning sessions. Yet, the presumed cortical activity that supported task-dependent reduction of H-reflex size during conditioning trials, did not lead to sustained reduction of the soleus H-reflex excitability in the long-run. This paradoxical increase in the control reflex size resembles the response of cSMC-lesioned rats to down-conditioning (20); when cSMC-lesioned rats were exposed to H-reflex down-conditioning, their H-reflex size increased significantly to 136% of the initial size. Since in rats, most of the conditioned reflex change is estimated to be accounted for by the long-term change (31), their resemblance makes sense. Why would reflex down-conditioning after cortical (or supraspinal) lesion lead to an increase in the excitability of the H-reflex pathway? A possible partial explanation may be found in the relation between the corticospinal connection and spasticity. Among individuals with clinically motor complete SCI, Sangari et al. (39) found that the motor evoked potentials (MEPs) to transcranial magnetic stimulation, which indicate the presence of cortical/corticospinal – motoneuron connection, are present only in individuals with spasticity, and MEP size is positively correlated with the severity of spasticity. Thus, one can imagine the possibility that when/if reflex down-conditioning increases cortical/corticospinal activity, it would also lead to increasing spastic hyperreflexia outside of the conditioning paradigm. Since we did not assess clinical spasticity after conditioning in this study, further investigation of this possibility would have to wait until a future study.

Perhaps, the most obvious question here would be what differed between the Ss and Sns groups, or what made the control reflex size to increase in the Sns group of individuals. As summarized in Table 1 and discussed above, age, stroke chronicity, mobility (ability to ambulate), or pre-conditioning H-reflex size does not differ between the Ss and Sns groups. Then, what would make the control reflex to change in different ways across different individuals? In this and other reflex conditioning studies, the control reflex that is measured outside of the operant conditioning paradigm reflects the state of ongoing negotiation among different motor skills and behaviors (including reflex conditioning trials) (7) on where to set the norm of the excitability of that reflex pathway. Thus, it is possible that some subtle differences in day-to-day reflex conditioning performance (i.e., the extent of task-dependent adaptation, see Table 2) or individuals' daily physical activity that was not captured in this study might have contributed to different long-term control reflex changes (both in magnitude and direction) across different individuals. Further studies are clearly needed to determine whether and to what extent the individual's neural, medical, physiological, and behavioral profiles prior to conditioning affects the control reflex plasticity, and how they may contribute to the spinal cord's negotiated equilibrium (7).

### Therapeutic implications

As the initial attempt to obtain a clue on whether or not there is a link between the reduction of H-reflex size and gait function improvement, the present study included the assessment of 10-m walking speed before and after 30 down-conditioning sessions. Ten-meter walking speed increased by various amount in individuals whose H-reflex became smaller (+0.14 m/s on average), and the participants with larger decreases in H-reflex size had relatively larger gain in walking speed. On the other hand, in individuals whose H-reflex size did not decrease, the walking speed did not decrease, supporting the unlikeliness of H-reflex down-conditioning causing detrimental effects on already impaired gait even when conditioning was not successful. Considering the variability in walking speed gain among individuals whose H-reflex size decreased, it is clear that the clinical and functional impact of H-reflex down-conditioning has to be investigated further in a larger cohort of post-stroke survivors in the future.

Operant conditioning is a powerful method for modifying a behavior based on its consequences (4–6, 40, 41). With operant conditioning of a spinal reflex, people learn and practice a simple skill of controlling/changing a spinal reflex behavior (6, 42), which leads to CNS multi-site plasticity beyond changes in the targeted reflex pathway (5, 42–45). When acquired and practiced over time, a new skill of producing a smaller soleus H-reflex in standing may help to improve walking in people with chronic incomplete SCI and spastic hyperreflexia (25). When the present groups of individuals after stroke were exposed to the H-reflex down-conditioning protocol, which was the same as the one used in the previous study in SCI (25), the conditioning success rate and time course of reflex changes differed from those in people with SCI, potentially due to the differences in lesion locations (i.e., spinal vs. supraspinal/cortical) and/or neural mechanisms of spasticity (46–52). Still, the soleus H-reflex down-conditioning did not negatively affect the impaired gait when the conditioning was not successful. Successful conditioning appeared to contribute to gait improvement. This delineates potential positive therapeutic effects of H-reflex down-conditioning. With reflex conditioning, one's innate (learning-related) plasticity is induced at the targeted neural pathway reflected in the size of a reflex; and then, through iterative and continuous “negotiation” with other neural behaviors, conditioning-induced plasticity may modify the function of that pathway over time (5, 7). In such process, non-beneficial changes are likely rejected or compensated for by other forms of plasticity. Thus, reflex conditioning wouldn't provide an instant miraculous fix, but its therapeutic effects would be non-negative.

## Conclusion

In this study, over the course of 30 down-conditioning sessions the soleus H-reflex size decreased in 50% of participants, supporting the feasibility of reflex down-conditioning in people after stroke. At the same time, the long-term plasticity development and conditioning success rate appeared to differ from those reported in individuals with SCI (25). This may reflect a critical role of supraspinal activity in producing long-term plasticity in the spinal cord, as previous animal studies suggested (19, 20). The present findings on the effects of H-reflex down-conditioning on walking speed may indicate potential positive therapeutic value of H-reflex down-conditioning in people after stroke.

## Data availability statement

The raw data supporting the conclusions of this article will be made available by the authors, without undue reservation.

## Ethics statement

The studies involving human participants were reviewed and approved by Helen Hayes Hospital and Medical University of South Carolina Institutional Review Board. The patients/participants provided their written informed consent to participate in this study.

## Author contributions

AT and RS conceived and designed research. AT, CG, and WF performed data collection. AT and CG analyzed data. AT, WF, and RS interpreted results. AT drafted manuscript. AT, CG, WF, and RS edited and revised manuscript. All authors approved the final version of manuscript.

## Funding

This work was supported in part by the National Institutes of Health (NIGMS) P20-GM109040 (PI: Kautz), U54-GM104941 (Institutional Development Award, PI: Binder-MacLeod), National Institute of Biomedical Imaging and Bioengineering (NIBIB) EB018783 (PI: Wolpaw), National Institute of Neurological Disorders and Stroke, U44 NS114420 (PI: Clements/Thompson/Wolpaw), and the New York State Spinal Cord Injury Research Trust C33279GG (PI: Wolpaw).

## Conflict of interest

The authors declare that the research was conducted in the absence of any commercial or financial relationships that could be construed as a potential conflict of interest.

## Publisher's note

All claims expressed in this article are solely those of the authors and do not necessarily represent those of their affiliated organizations, or those of the publisher, the editors and the reviewers. Any product that may be evaluated in this article, or claim that may be made by its manufacturer, is not guaranteed or endorsed by the publisher.
